# The Dram-Grain Plan of Writing Prescriptions

**Published:** 1888-09

**Authors:** C. H. Merrick

**Affiliations:** Washington Territory


### THE DRAM-GRAIN PLAN OF WRITING PRESCRIPTIONS.

BY C. H. MERRICK, M. D., WASHINGTON TERRITORY.

I would like to tell your readers how to write prescriptions by the above named plan. Suppose I want an eight-ounce mixture. Instead of the ℞ I write 64 in plain figures. This indicates that there are 64 teaspoonful doses in the prescription. Now I write down in regular order the names of the medicines I want in the prescription. To illustrate: I will name the medicines a. b. c. etc. I want to give the patient at each teaspoonful dose 10 grains of a, 15 grains of b, 6 grains of c, and 1 grain of d. I write:

64. A......................................10.00,

B......................................15.00,

C......................................6.00,

D......................................1.00.

Dose.—Teaspoonful every four hours, etc.

The 64 indicates to the druggist that an eight ounce mixture is called for. The figures call for drams, that is, 10 drams of a, 15 of b, etc. The druggist, after putting in the various medicines, will add any suitable vehicle to make a total of eight ounces.

Discard all signs and write drams and grains just as we write dollars and cents. I call an eight-ounce mixture a full sized prescription, because the figures placed at each medicine, calling for drams, expressed grains to each teaspoonful dose.

Suppose I want a three-quarter sized prescription. I write:

48. A.................................... 9.00,
B....................................15.00,
C.................................... 6.00,
D.................................... 3.00.

Dose.—Teaspoonful, etc.

The figures in this prescription, calling for drams, are just three-quarters of the grains or minims I want in each dose. Example: I want to give twelve minims of a, so I write three-fourths of twelve for the druggist to put up in drams.

One more example will certainly be enough to illustrate the system.

Suppose I want a four ounce mixture. I want to give of a 10 grains, of b 4 grains, of c 12 minims, and of d 20 minims. This is a half sized prescription, consequently I write half the amount expressing drams I want in grains or minims to each teaspoonful dose. I write:

32. A.................................... 5.00,

B.................................... 2.00.

C.................................... 6.00,

D....................................10.00.

Dose.—Teaspoonful, etc.

How about fraction of grains? Simplest thing in the world. Suppose in a 64 prescription I want half a grain of a, quarter of a grain of b, a thirty-second of a grain of c, and an eighth of a grain of d, I write:

64. A....................................32,
B....................................16,
C.................................... 2,
D.................................... 8.

Dose.—Teaspoonful, etc.

Remember figures to the left of a line or point express drams, those to the right call for grains or minims. To find out what you want the rule is plain. Divide the symbol, that is the figures expressing the size of the prescription, by the fraction of a grain wanted, and write the result for the druggist to furnish in grains.

Once more. I want a four ounce mixture. I want of a 10 grains, of b ½ a grain, of c 1 grain, of d .32 of a grain to each dose. I write:

| | | |
|---|---|---|
| 32 | A.......................................... | 5.00, |
| | B.......................................... | .16, |
| | C.......................................... | .32, |
| | D.......................................... | .01. |

Dose.—Teaspoonful etc.

These rules apply to all sized prescriptions, and ten minutes thought will fasten the whole plan in the mind. If tablespoonful doses are wanted, it is an easy thing to calculate accordingly. For instance, in a 32 prescription eight drams of a in teaspoonful doses are the same as quarter of eight drams in tablespoonful doses. Rule: calculate for teaspoonful doses, then if tablespoonful doses are ordered write one-fourth for the druggist to furnish.

I shall be pleased to explain any point not clearly expressed in this hurriedly written article.

